# Supplementary material for: De novo gene signature identification from single‐cell RNA‐seq with hierarchical Poisson factorization
Source: Mol Syst Biol. 2019 Feb 22;15(2):e8557. doi: 10.15252/msb.20188557 (PMC6386217; doi:10.15252/msb.20188557)
Supplement: Supplementary file 1 — Appendix [file MSB-15-e8557-s001.pdf]

Appendix for "*De Novo* Gene Signature Identification from Single-Cell RNA-Seq with Hierarchical Poisson Factorization"

Table of Contents

Appendix Table S1
Appendix Figures S1-S8

	**PBMCs**	**Matcovitch *et al.***	**TS543**	**HGG**
**scRNA-seq platform**	10x Chromium	MARS-seq	Yuan & Sims	Yuan & Sims
**Number of cells**	4340	3456	9924	3109 core<br>3000 margin
**Min. cells expressing gene (for filtering)**	5	5	10	10
**Number of protein-coding genes after filtering**	13030	8086	11807	14730
**Sparsity of filtered data**	90%	94%	92%	93%
**Number of factors (K)**	10	10	5	14

Appendix Table S1: Datasets and parameters used.

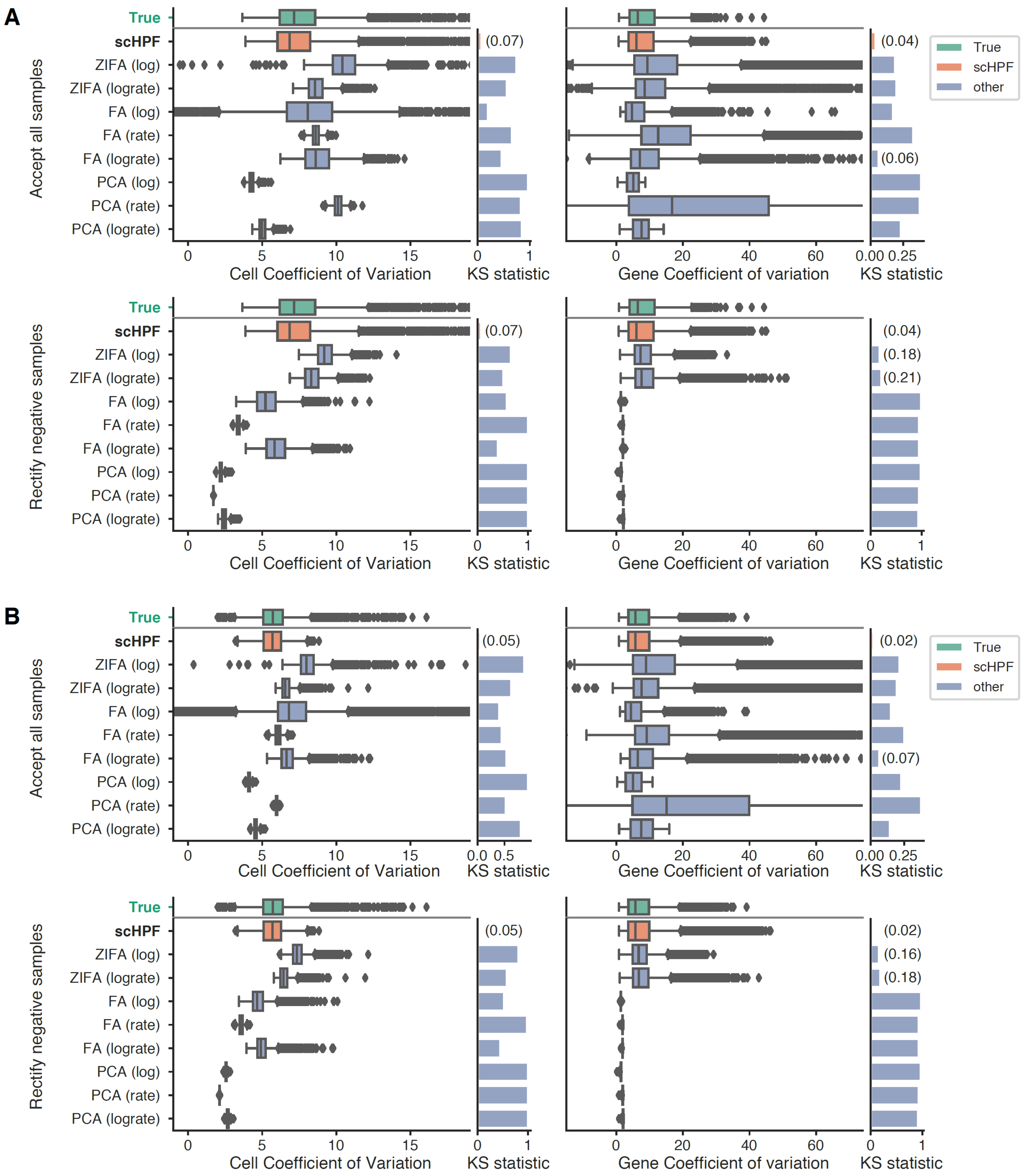

Appendix Figure S1: Same as Figure 2B-C, but for **(A)** Matcovitch *et al*. and **(B)** TS543. X-axes limits for boxplots are set to include all coefficients of variation from the true distribution and scHPF, and as many coefficients of variation from other methods as possible.

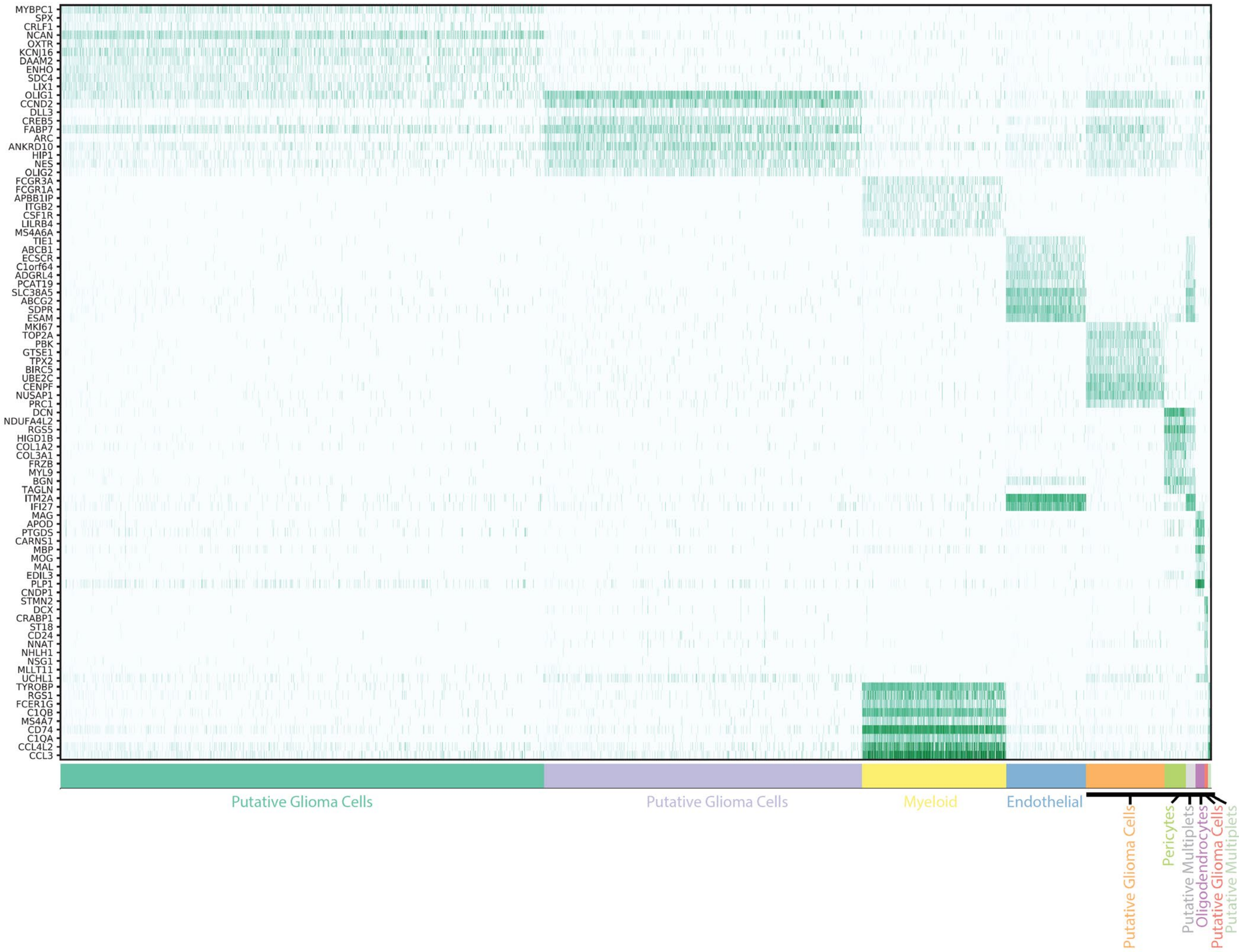

Appendix Figure S2: Heatmap gene expression in a high-grade glioma with cells (columns) ordered by Louvain cluster (Methods) and genes (rows) selected as the top ten most specific genes in each cluster. Bottom color bar shows clusters and putative labels based on expression of canonical marker genes and aneuploidy analysis (see Figure S5).

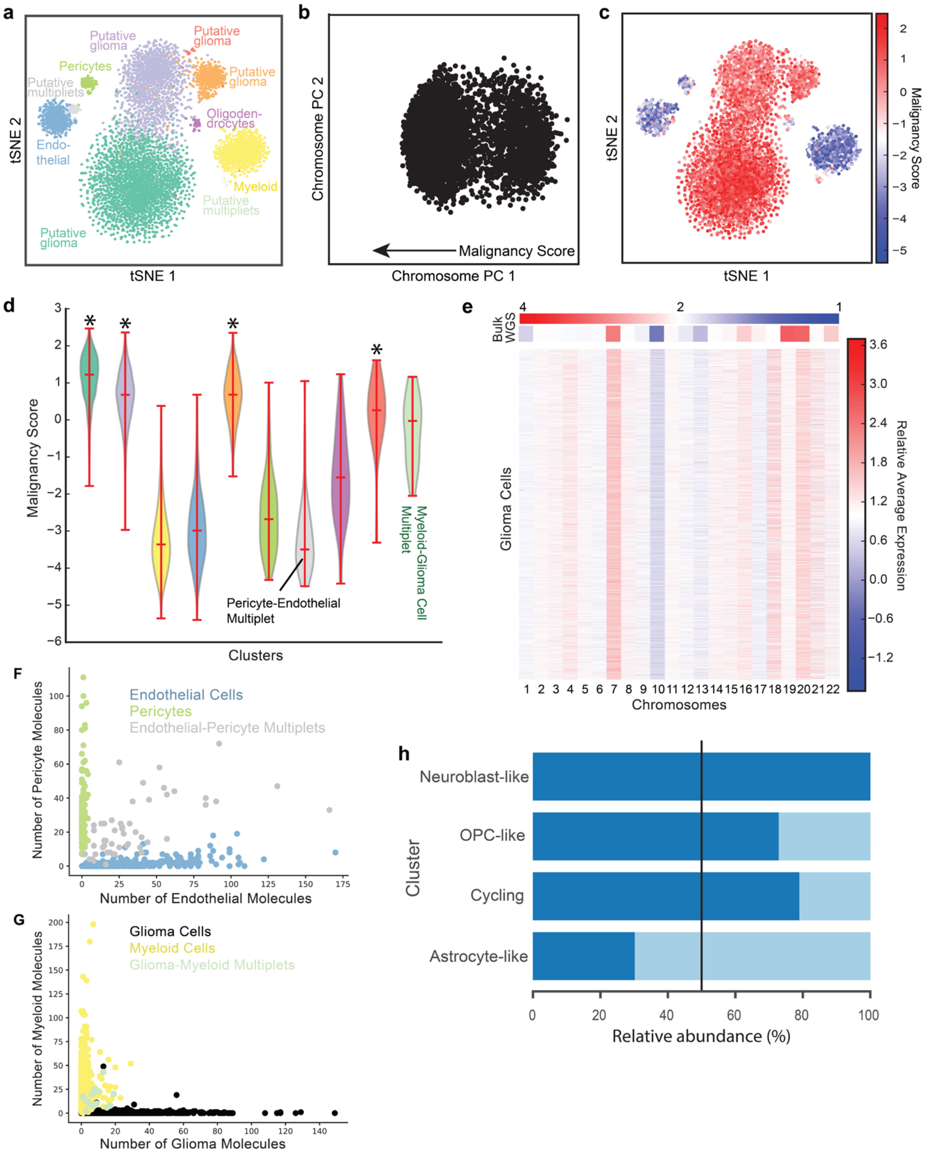

Appendix Figure S3: (A) t-distributed Stochastic Neighbor Embedding (tSNE) (Maaten & Hinton, 2008) plot of tumor cells, labeled by cluster (also see figure S4). **(B)** PCA of whole-chromosome expression for each cell. The first principle component (PC1), which we call a malignancy score, separates putative glioma from non-malignant cells. **(C)** tSNE plot of all cells, colored by malignancy score. **(D)** Violin plots of malignancy scores for each cluster. Putative glioma clusters are starred. **(E)** Main heatmap shows putative glioma cells' (rows) relative average expression of each chromosome (columns). Values generally agree with bulk whole genome sequencing (WGS) of the tumor (top heatmap). **(F)** Barnyard plot of cells in the endothelial (blue), pericyte (green) or endothelial-pericyte multiplet (gray) clusters. Total number of molecules for the ten most endothelial-specific genes by a binomial test are on the x-axis, and total number of molecules for the top ten most pericyte-specific genes are on the y-axis. **(G)** Barnyard plot of all putative glioma cells (black), cells in the myeloid cluster (yellow), and cells in the putative myeloid-glioma multiplet cluster (green). Total number of molecules of the ten most glioma-specific genes by a binomial test are on the x-axis, and total number of molecules of the ten most myeloid-specific genes are on the y-axis. **(H)** Relative abundance of glioma subpopulations in the core (navy) and margin (light blue).

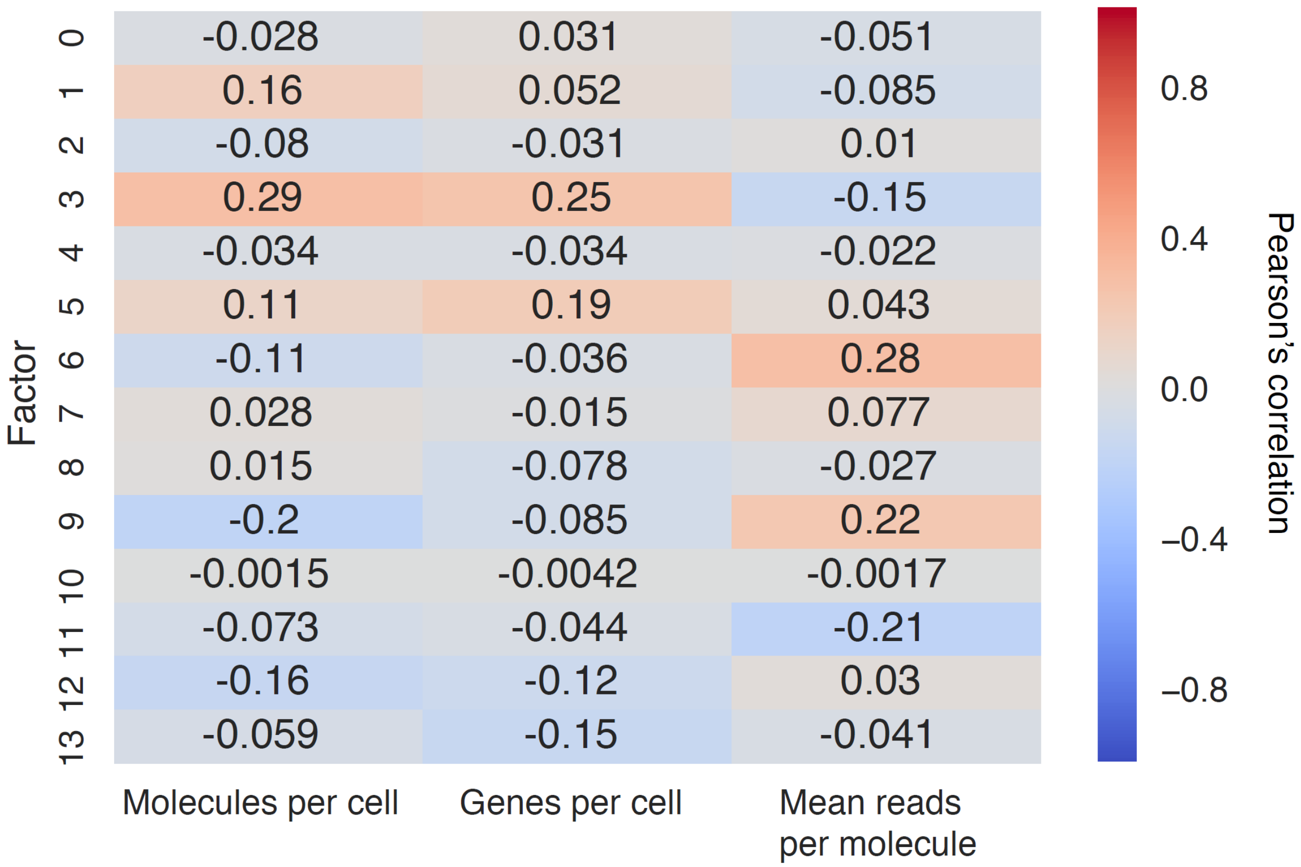

Appendix Figure S4: scHPF cell scores are largely uncorrelated with technical variables. Some factors modestly correlated with variables like molecules per cell and genes per cell are associated with physically larger cell types such as endothelial cells (factor 3) or dividing cells (factor 5).

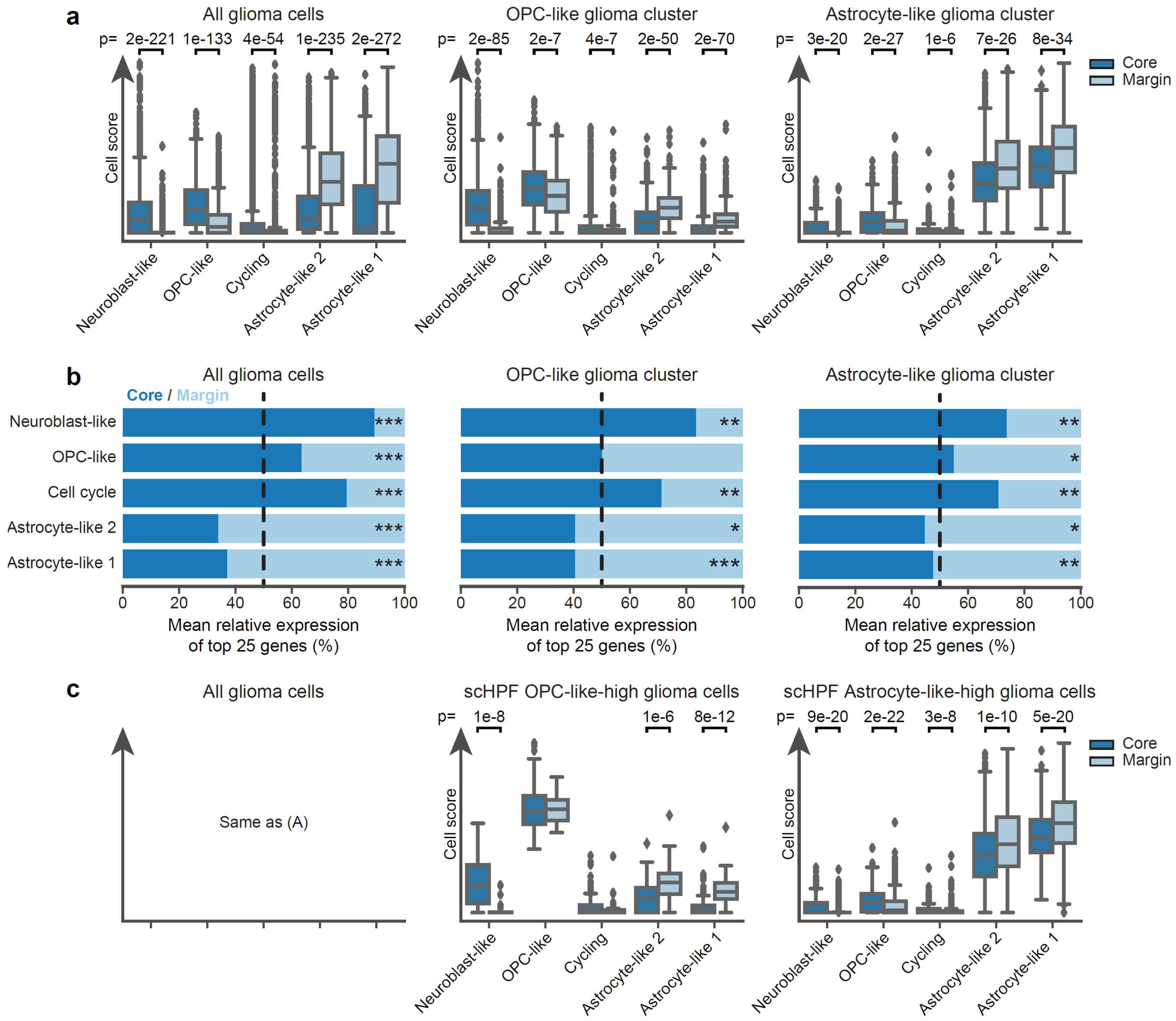

Appendix Figure S5: (A) Boxplots of scHPF cell scores for all glioma cells (left), OPC-like glioma cells (center), and astrocyte-like glioma cells (right) show strong regional bias towards the core (navy) or margin (light blue). Bracketed values show Bonferroni-corrected p-values from the Mann-Whitney U-test for the difference between two distributions. **(B)** Program scores, derived as the mean relative expression of the top 25 genes in each factor, recapitulate cell scores' regional biases. $*** = p < 10^{-50}$, $** = p < 10^{-10}$, $* = p < 10^{-2}$. All p-values are Bonferroni corrected. Expression values were converted to counts per median and log10 scaled before averaging. **(C)** Same as (A), but with OPC-like and astrocyte-like glioma subpopulations defined as cells with maximal scHPF cell scores in the OPC-like factor or one of the two astrocyte-like factors, respectively.

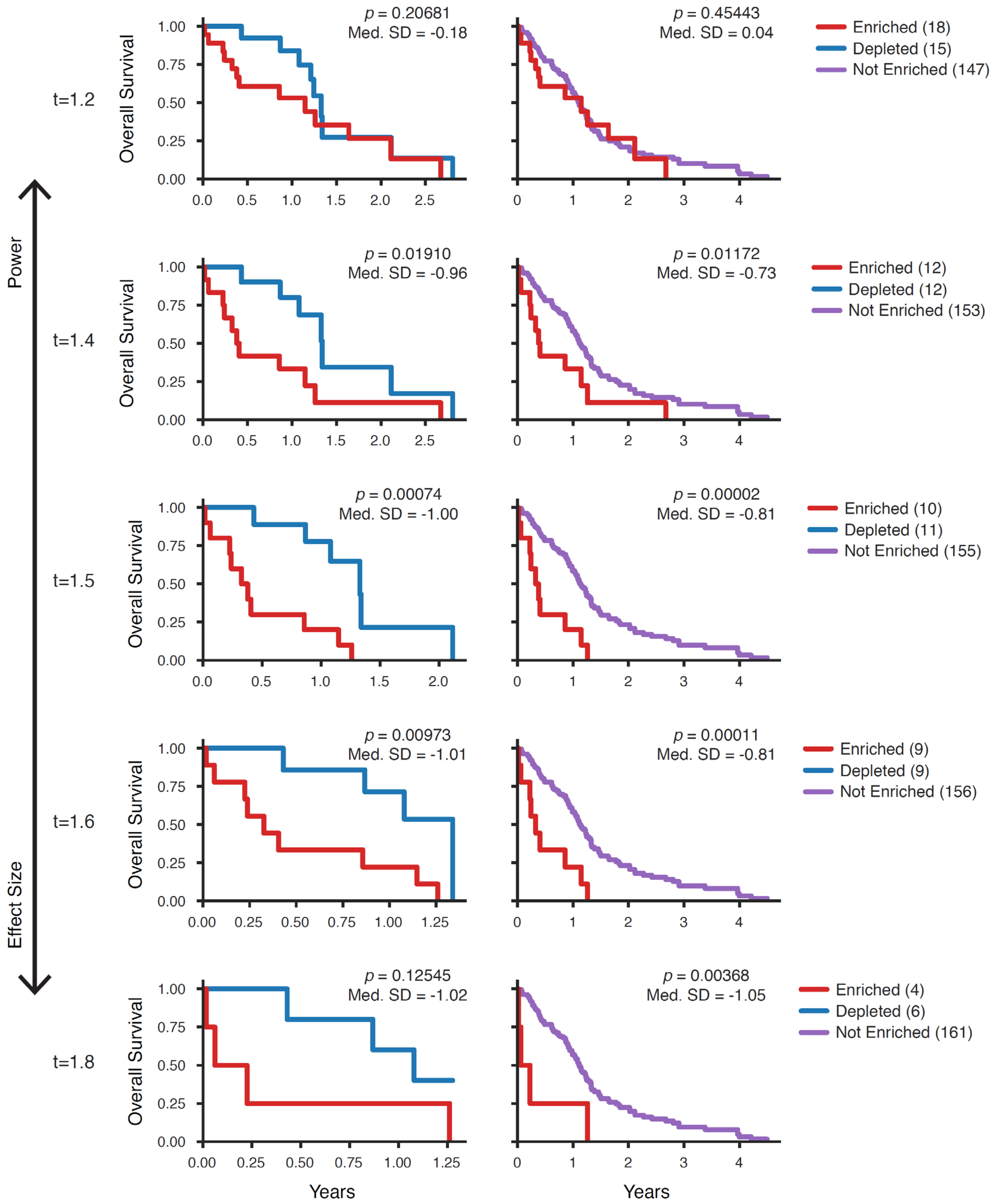

Appendix Figure S6: Kaplan-Meier curves show survival differences in TCGA for donors enriched (red), not enriched (purple), and depleted (blue) for the 25 top scoring genes in astrocyte-like factor 1 (Methods) at different effect size cutoffs. Median survival difference (Med. SD) increases as the effect size cutoff (t) for inclusion in enriched and depleted cohorts increases. Statistical power decreases as effect size increases and treatment groups become smaller.

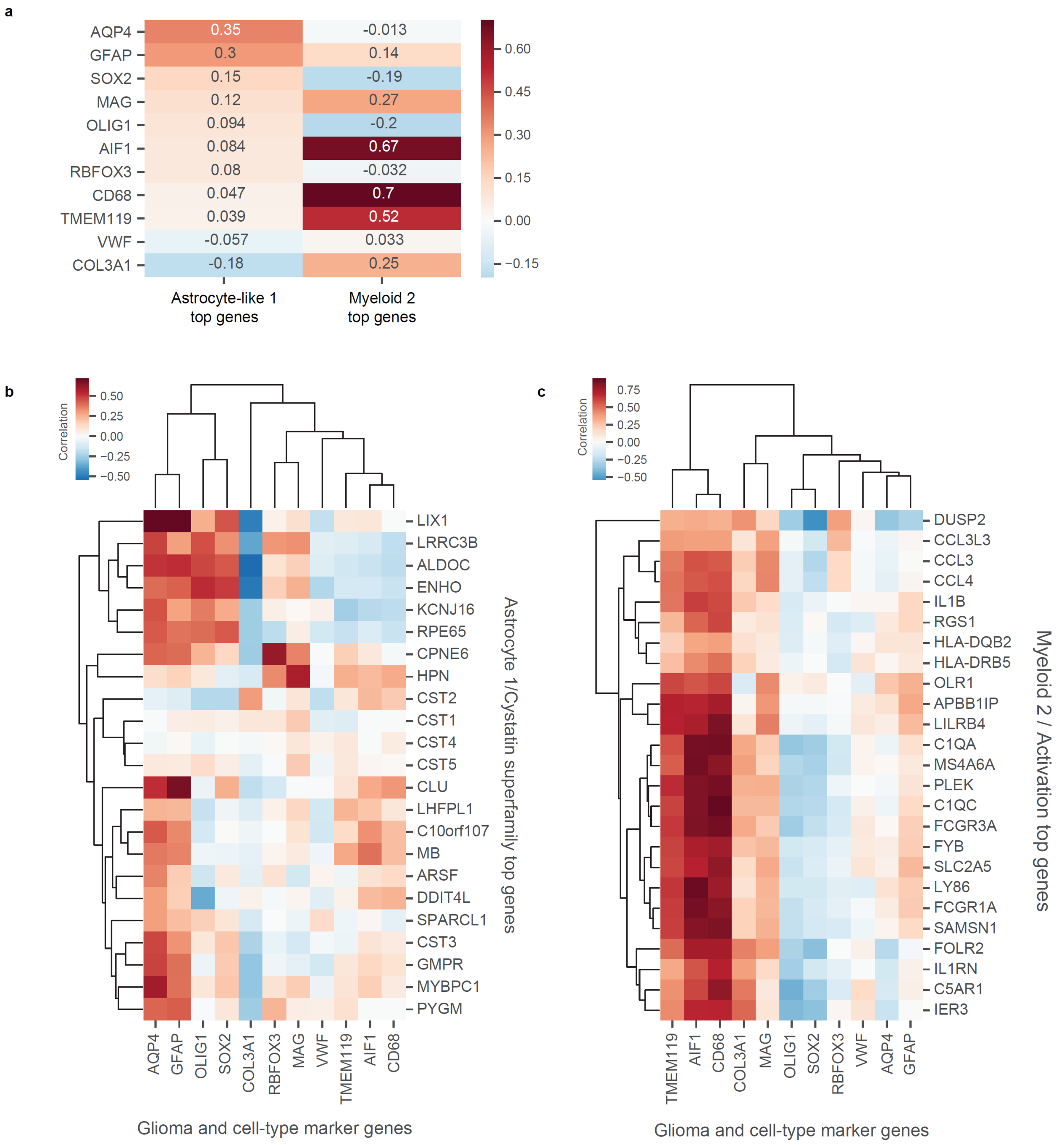

Appendix Figure S7: (A) Median correlation of the top 25 genes from two scHPF factors with glioma and cell type marker genes in TCGA GBM RNA-seq. scHPF Astrocyte-like 1 is best correlated the GBM-specific marker, SOX2, and astrocyte markers. In contrast, scHPF Myeloid 2 is best correlated with microglial/macrophage markers. **(B & C)** Hierarchically clustered correlation of marker genes with the top 25 genes from scHPF Astrocyte-like 1 (B) and scHPF Myeloid 2 (C).

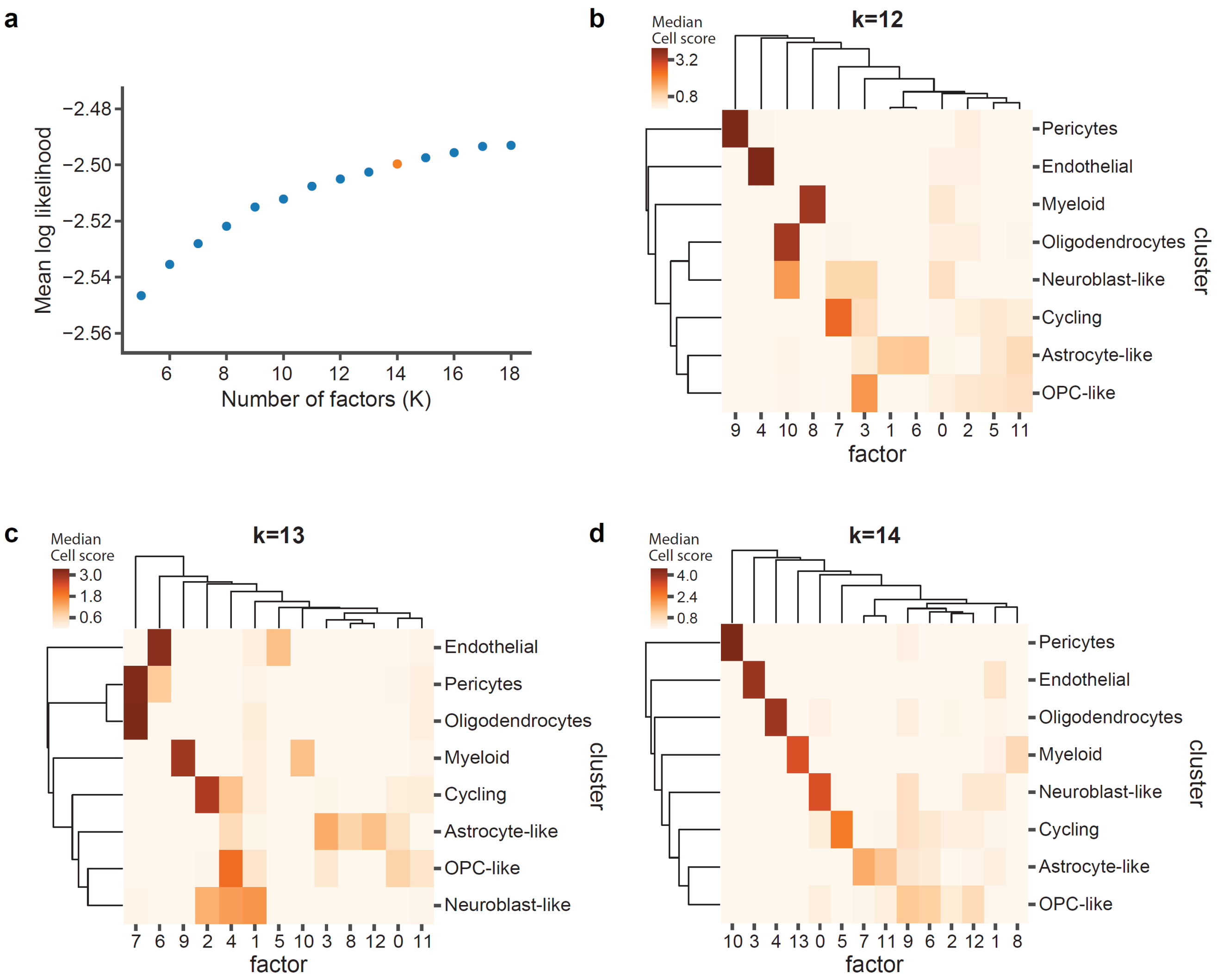

Appendix Figure S8: (A) Mean log likelihood for scHPF of a high-grade glioma at different values of *K* (higher is better). **(B-D)** Median factor score in each cluster at 12, 13, and 14 factors. With 12 factors (B), oligodendrocytes and neuroblast-like cells are both most closely associated with the same factor. Similarly, with *K*=13 (C), oligodendrocytes and pericytes are both most closely associated with the same factor. At *K*=14 (D), all clusters are most closely associated with at least one unique factor.
