# Supplementary material for: De novo gene signature identification from single‐cell RNA‐seq with hierarchical Poisson factorization
Source: Mol Syst Biol. 2019 Feb 22;15(2):e8557. doi: 10.15252/msb.20188557 (PMC6386217; doi:10.15252/msb.20188557)
Supplement: Supplementary file 2 — Expanded View Figures PDF [file MSB-15-e8557-s002.pdf]

Expanded View Figures

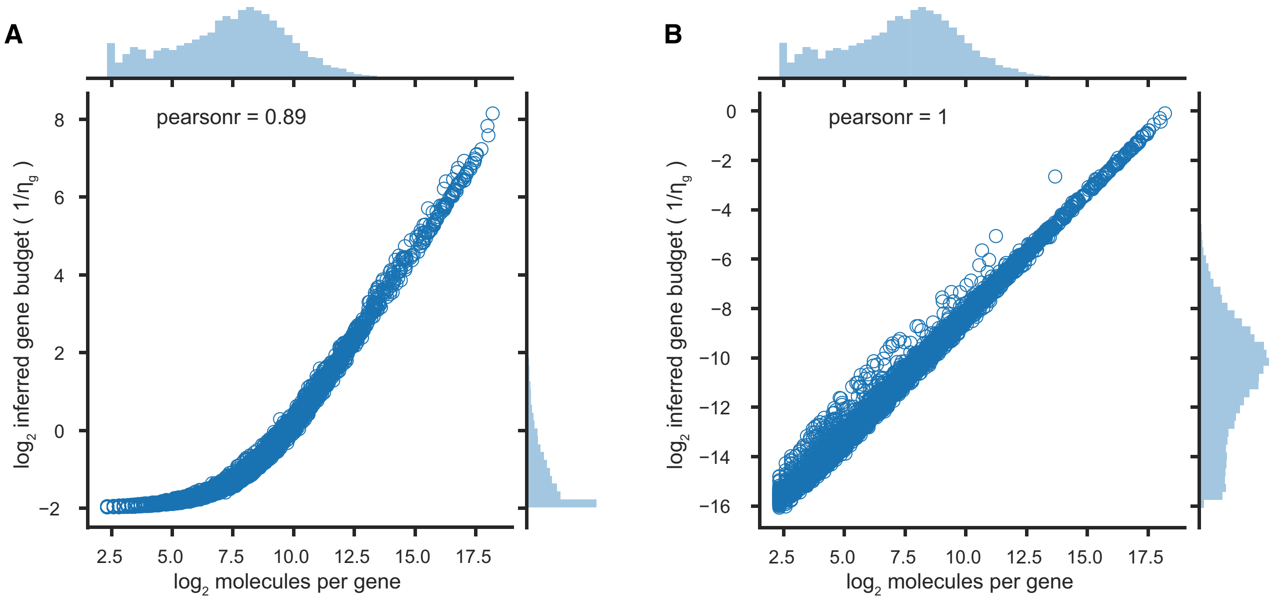

Figure EV1. Effect of emperically set hyperparemeters on inference of budgets.

A, B Scatter plots of log2 molecules per gene (x-axes) versus the log2 inferred gene budgets (*y*-axes), with hyperparameters (A) a', b', c' and d' set to 1 or (B) determined empirically in a representative experiment on peripheral blood mononuclear cells. Histograms on top and right show the marginal probability distributions along each axis.

© 2019 The Authors

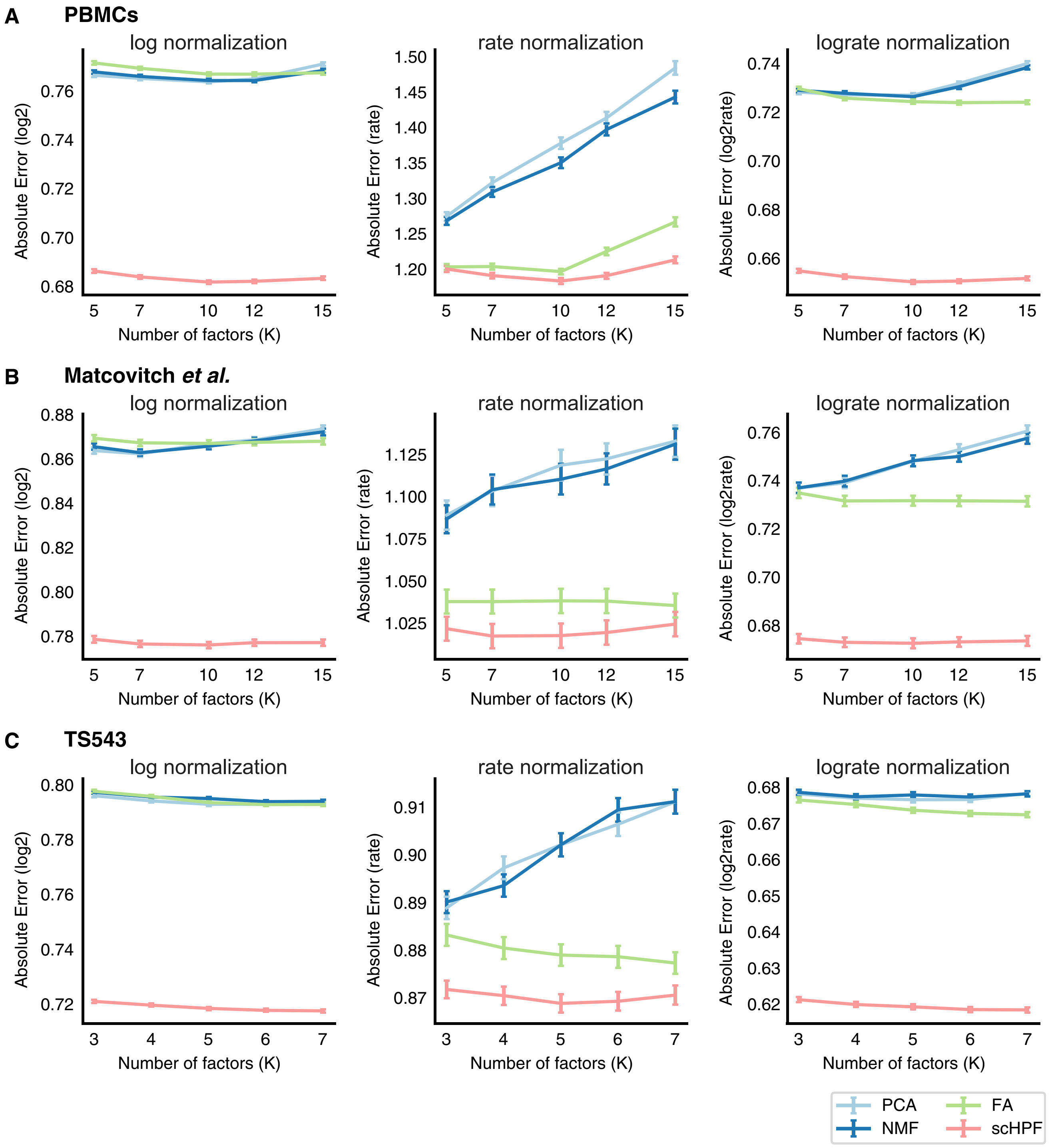

Figure EV2. Predictive performance across different number of factors.

A–C scHPF has lower test error than other method and normalization combinations on a withheld partition of the (A) PBMC, (B) Matcovitch *et al*, and (C) TS543 datasets for several different numbers of factors. Error bars show standard error of the mean across all withheld values (4% of non-zero matrix entries randomly selected from each dataset: 220391 for PBMCs, 68895 for Matcovitch *et al*, 356241 for TS543); center values show the mean. scHPF's predictions were normalized before calculating error.

 © 2019 The Authors

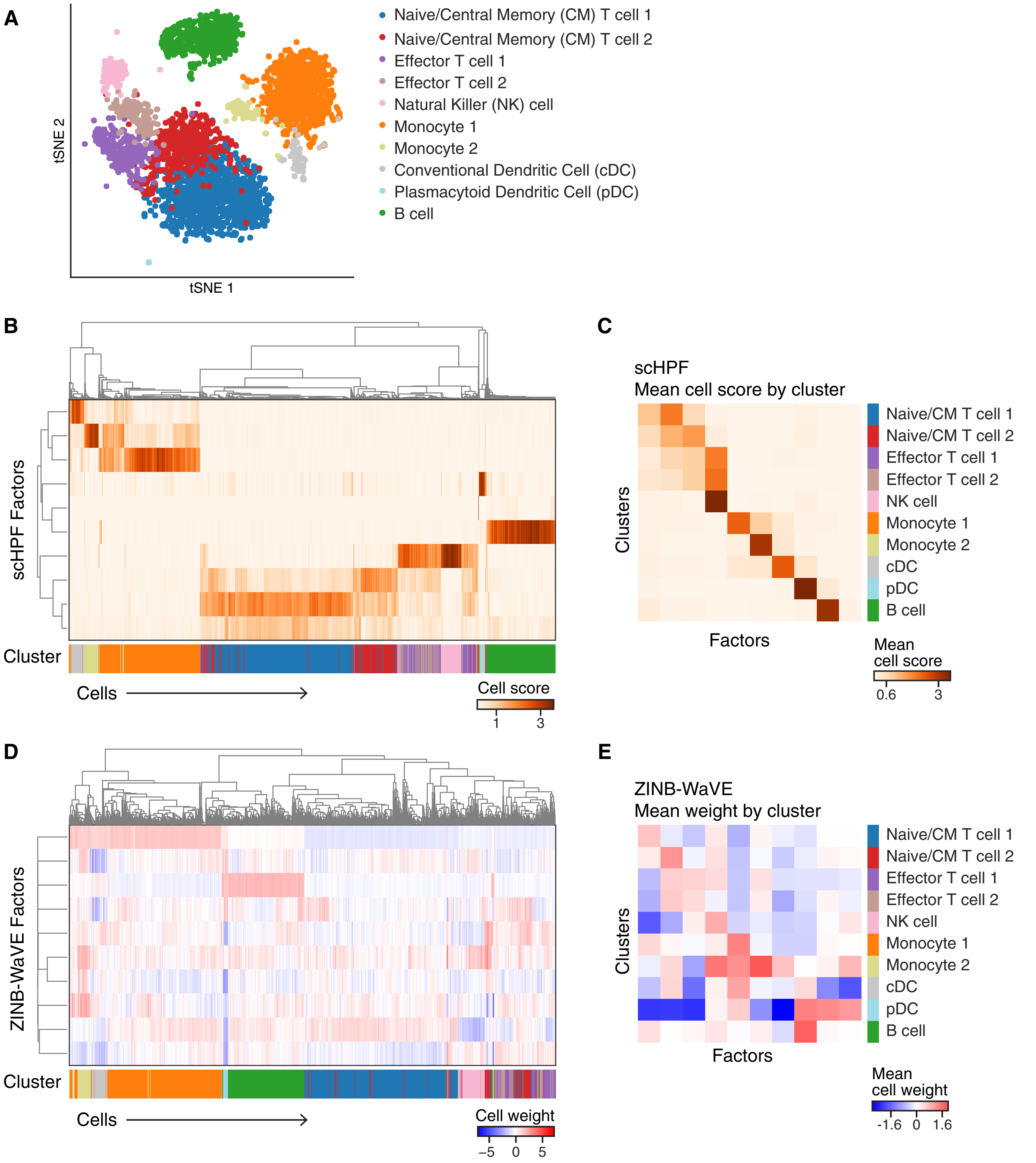

Figure EV3. Cell-type associations of factors obtained with scHPF or ZINB-WaVE on the PBMC data.

A UMAP embedding of PBMCs, colored by cluster.

B Main heatmap shows average-linkage hierarchical clustering of scHPF cell scores for each factor (rows) and cell (columns). Bottom colorbar shows cells' assigned cluster in (A).

C scHPF factors' (columns') mean cell score for each cluster (row).

D, E Same as (B, C), but for ZINB-WaVE cell weights.

© 2019 The Authors

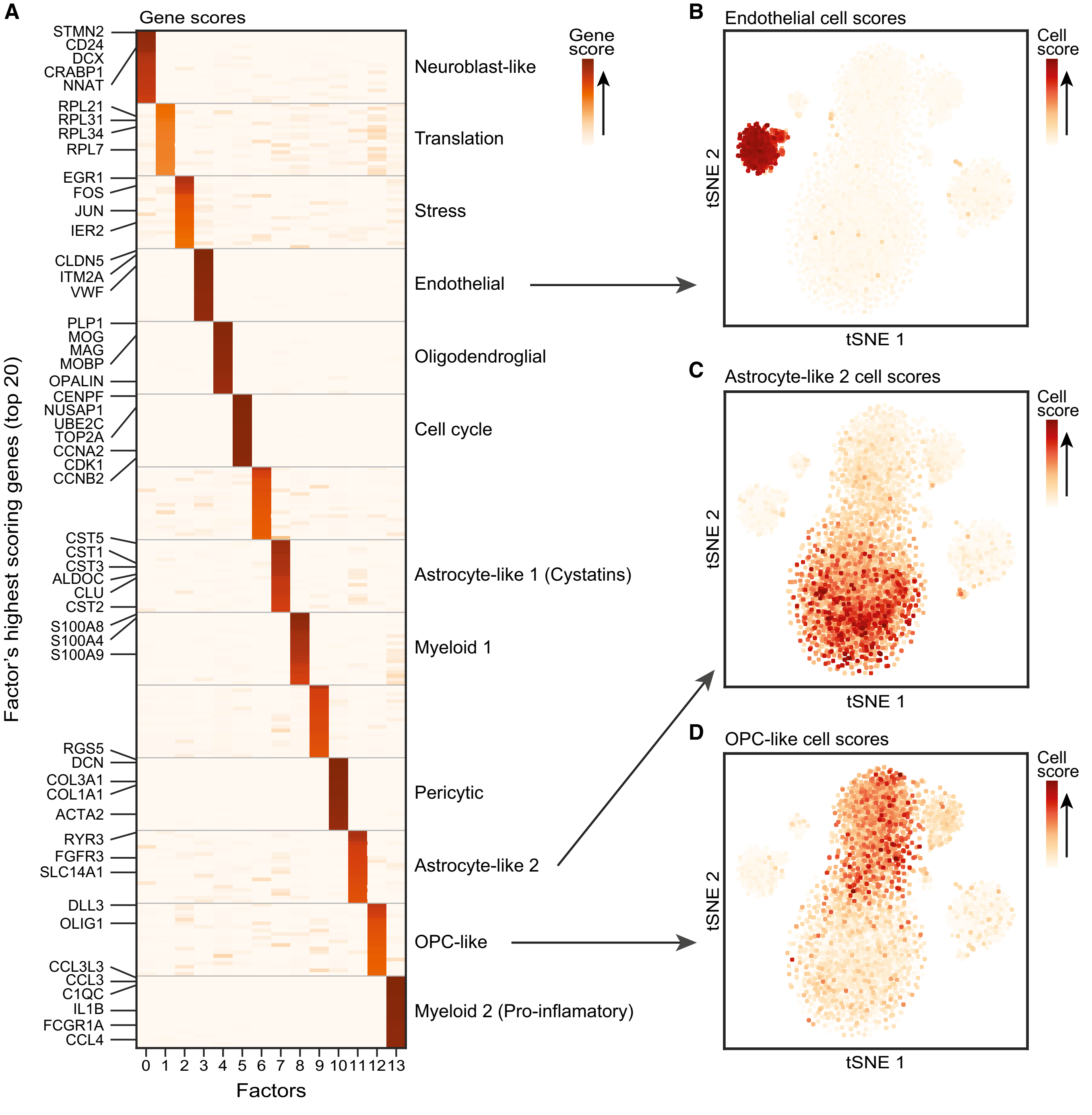

Figure EV4. scHPF discovers gene signatures in a high-grade glioma.

A Heatmap of scHPF gene scores for each factor (columns) and the top twenty genes per factor (rows). Canonical marker genes and genes from a protein superfamily are highlighted.

B–D tSNE of all cells colored by their scHPF cell scores for a factor that marks a discrete population of endothelial cells (B), one of two glioma-associated factors that highly ranks astrocyte marker genes (C), and a glioma-associated factor that highly ranks OPC marker genes.

© 2019 The Authors

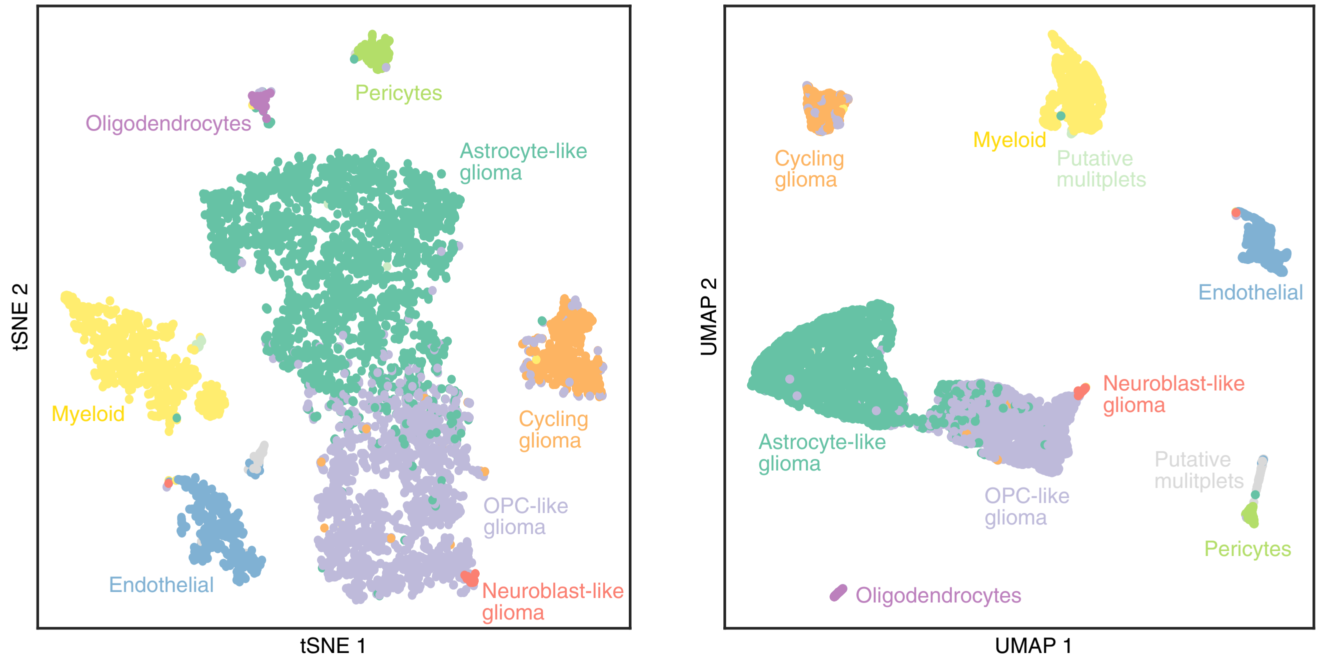

Figure EV5. scHPF cell scores can be used as a low-dimensional input to visualization algorithms.

tSNE (left) and UMAP (right) embeddings of HGG cells using scHPF cell scores as a low-dimensional input. Pearson's correlation distance was used as a distance metric.

© 2019 The Authors
